# Inequity in uptake of hospital-based childbirth care in rural Tanzania: analysis of the 2015–16 Tanzania Demographic and Health Survey

**DOI:** 10.1093/heapol/czab079

**Published:** 2021-07-19

**Authors:** Manuela Straneo, Lenka Benova, Claudia Hanson, Piera Fogliati, Andrea B Pembe, Tom Smekens, Thomas van den Akker

**Affiliations:** Athena Institute, VU Amsterdam, De Boelelaan 1085, 1081 HV Amsterdam, The Netherlands; Sexual and Reproductive Health Group, Department of Public Health, Institute of Tropical Medicine, Nationalestraat 155, 2000 Antwerp, Belgium; Faculty of Epidemiology and Population Health, LSHTM, Keppel Street, London WC1E 7HT, UK; Karolinska Institutet, 171 77 Stockholm, Sweden; Faculty of Infectious and Tropical Diseases, LSHTM, Keppel Street, London WC1E 7HT, UK; Doctors with Africa-CUAMM, Av. Mártires da Machava n.º 859 R/C, Cidade de Maputo, Moçambique; Department of Obstetrics and Gynecology, Muhimbili University of Helath and Allied Sciences, PO Box 65001, Dar es Salaam, United Republic of Tanzania; Department of Public Health, Institute of Tropical Medicine, Nationalestraat 155, 2000 Antwerp, Belgium; Athena Institute, VU Amsterdam, De Boelelaan 1085, 1081 HV Amsterdam, The Netherlands; Department of obstetrics and Gynecology, Leiden University Medical Center, Rapenburg 70, 2311 EZ Leiden, The Netherlands

**Keywords:** Obstetrics, maternal and child health, maternal services, equity, primary health care, health inequalities, poverty, rural, hospital, health facilities, health care utilization

## Abstract

Proportions of facility births are increasing throughout sub-Saharan Africa, but obstetric services vary within the health system. In Tanzania, advanced management of childbirth complications (comprehensive emergency obstetric care) is offered in hospitals, while in frontline, primary health care (PHC) facilities (health centres and dispensaries) mostly only routine childbirth care is available. With over half (54%) of rural births in facilities, we hypothesized the presence of socio-economic inequity in hospital-based childbirth uptake in rural Tanzania and explored whether this relationship was modified by parity. This inequity may compound the burden of greater mortality among the poorest women and their babies. Records for 4456 rural women from the 2015–16 Tanzania Demographic and Health Survey with a live birth in the preceding 5 years were examined. Proportions of births at each location (home/PHC/hospital) were calculated by demographic and obstetric characteristics. Multinomial logistic regression was used to obtain crude and adjusted odds ratios of home/PHC and hospital/PHC births based on household wealth, including interaction between wealth and parity. Post-estimation margins analysis was applied to estimate childbirth location by wealth and parity. Hospital-based childbirth uptake was inequitable. The gap between poorest and richest was less pronounced at first birth. Hospital-based care utilization was lowest (around 10%) among the poorest multiparous women, with no increase at high parity (≥5) despite higher risk. PHC-based childbirth care was used by a consistent proportion of women after the first birth (range 30–51%). The poorest women utilized it at intermediate parity, but at parity ≥5 mostly gave birth at home. In an effort to provide effective childbirth care to all women, context-specific strategies are required to improve hospital-based care use, and poor, rural, high parity women are a particularly vulnerable group that requires specific attention. Improving childbirth care in PHC and strengthening referral linkages would benefit a considerable proportion of women.

**Key messages**

- The proportion of facility births is increasing in rural Tanzania, but obstetric care at different levels of the health system varies. We hypothesized a differential use of hospitals for childbirth and assessed interaction between wealth and parity. Inequity in hospital-based childbirth care use may contribute to the burden of greater mortality among the poorest women and their babies.
- Hospital-based childbirth care use was inequitable among women in rural Tanzania. The gap between the poorest and richest use was less pronounced among women at first parity. Uptake of hospital-based care was lowest (around 10%) among poorest multiparous women, remaining low at high parity (≥5) despite higher risk of complications and death.
- Rural women’s use of PHC childbirth care after the first birth was noteworthy, ranging between 30 and 51% depending on wealth and parity.
- As part of efforts to reach all women with appropriate, timely care, strategies are required to improve uptake of hospital-based care particularly among poor, rural, high parity ones. Improving quality of childbirth care in PHC and referral linkages would benefit a substantial proportion of women.

## Introduction

Mortality around the time of childbirth is essentially a disease of poverty. An inverse relationship between poverty and maternal health has been known for over a century. Wide inequities in maternal and perinatal mortality exist between nations, with low- and middle-income countries being the most affected (Graham *et al.*, 2016). Sub-Saharan Africa (SSA), with only 14% of global population, accounted for 66% (201 000) of maternal deaths, 40% (1 027 000) of newborn deaths and 31% (1 060 000) of stillbirths in 2015 (Alkema *et al.*, 2016; Blencowe *et al.*, 2016; World Bank, 2020; WHO, 2015a). Wide gradients also exist within countries, with the poorest disproportionately affected (Ronsmans and Graham, 2006; Houweling *et al.*, 2007; Filippi *et al.*, 2016). Such inequities are often masked by national averages (Kinney *et al.*, 2010).

Providing effective childbirth care is challenging where resources are limited, and rural SSA is a particularly arduous setting (Campbell *et al.*, 2016). In SSA countries, primary health care (PHC) has been a central strategy to ensure access to services, including intrapartum care, for rural populations. Tanzania, with a population of 59 million (2020) (World Bank, 2020), has been at the forefront of PHC development after independence with its founding principles set out in the Arusha Declaration in 1967 (Bustreo *et al.*, 2019; Dominicus and Akamatsu, 1989). A vision of high-quality PHC for all is expressed in current policy (Vision 2025) (Tanzania URO, 2000). PHC facilities are dispensaries at village level and health centres at ward level (Tanzania MoHSW, 2015). Women can give birth at all levels of the health system, and childbirth in PHC facilities is encouraged for women with no known risk factors at onset of labour (Hanson *et al.*, 2015). In spite of a capillary PHC network, with 85% of the population living within 5 km from a facility, Tanzania’s maternal mortality ratio in 2017 remained high at 524 per 100 000 live births (a reduction of 39% from 2000) (WHO, 2015b; 2019) and was among 10 countries worldwide with the highest absolute numbers of maternal, newborn deaths and stillbirths (Lawn *et al*., 2016).

Obstetric care at different levels of the health system varies markedly in SSA (Campbell *et al.*, 2016). There is growing evidence that outcomes for mothers and their babies improve when women give birth in units offering high-quality care, not just in any facility (Gabrysch *et al.*, 2019; Hanson *et al.*, 2015; Lohela *et al.*, 2019). In Tanzania, advanced management of childbirth complications (including surgery and blood transfusions, equivalent to comprehensive emergency obstetric care [EmOC]) is available in hospitals, while lower-level, PHC facilities are generally able to provide routine childbirth care only (Campbell *et al.*, 2016; Kruk *et al.*, 2016). Although dispensaries and health centres differ in size, number of beds and staffing levels, both types of facilities have similar obstetric capacity and often do not reach a description of basic EmOC facility (Campbell *et al.*, 2016; Hanson *et al.*, 2013). Challenges to the provision of high-quality obstetric care in lower-level facilities in Tanzania have been amply described and include insufficient staffing, poor infrastructure and low birth volumes (Hanson *et al.*, 2013; Benova *et al.*, 2014; Kruk *et al.*, 2014; Hanson *et al.*, 2015; Straneo *et al.*, 2014; Baker *et al.*, 2015). Although national efforts are underway to increase obstetric care in health centres up to comprehensive EmOC, at the time of analysis very few had been upgraded. Higher mortality among poorer women and their babies may be compounded by their reduced hospital-based childbirth care uptake, where higher-quality obstetric care is more commonly found.

Over the past decade, a shift from home to facility births has been described in SSA, with increasing proportions of women giving birth in facilities in rural and urban contexts and across wealth groups, and Tanzania is no exception (Montagu *et al.*, 2017; Doctor *et al.*, 2019). However, strong socio-demographic differentials continue to be reported (Kyei-Nimakoh *et al.*, 2017; Moyer and Mustafa, 2013; Gabrysch and Campbell, 2009; Campbell *et al.*, 2016; Virgo *et al.*, 2017; Dunlop *et al.*, 2018). There is limited information on how socio-economic groups uptake obstetric care at different levels of the health system. Within the background of a renewed discussion of the most efficient configuration of childbirth care from an equity, quality and cost-efficiency perspective (Kruk *et al.*, 2016; Hanson and Schellenberg, 2019; Kruk *et al.*, 2018; Roder-DeWan, 2020), we aimed to estimate the levels of use of hospital-based childbirth care in rural Tanzania and its association with women’s socio-economic status. Given the association of poverty and high parity, and the latter’s implications for obstetric care, we investigated whether the association between wealth and hospital-based childbirth depended on parity.

## Methods

### Study setting

In Tanzania, the most recent (2015–16) Demographic and Health Survey (DHS) estimated that 63% of births in the 5-year period preceding the survey took place in health facilities (54% in rural and 86% in urban areas) (Tanzania MoHCDGEC, 2016). In the same period, there were 6790 facilities (including public, faith-based, parastatal and private), from which 257 (3.8%) were hospitals (Tanzania MoHSW, MoHMZ, National Bureau of Statistics [NBS], Office of the Chief Government Statistician [OCGS], and ICF, 2015). There were an estimated 12 million women of reproductive age and approximately 1.9 million births in 2015.

### Data and population

Data from the 2015–16 Tanzania DHS were used. DHS are cross-sectional, nationally representative surveys of households, with women of reproductive age (15–49 years) self-reporting on the use of reproductive and maternal healthcare. Approximately 12 500 households were visited, and 13 000 women interviewed. Records of women living in rural areas in mainland Tanzania were used in this analysis, if they reported a live birth in the 5 years preceding the survey. Classification as ‘rural’ in DHS was based on census enumeration units (Tanzania NBS and Zanzibar OGCS, 2013).

### Definitions

The outcome variable was the location of the most recent live birth, in three categories: home (respondent’s home, other home and en route to provider), PHC facility (dispensary, health centre, maternity home and ‘other facility’) and hospital (district, regional, referral or tertiary/university). All public and private (non-profit/profit) PHC facilities and hospitals were included.

*Socio-economic status (SES)*: In DHS, SES is based on availability of durable household assets (Vyas and Kumaranayake, 2006). A wealth score is generated for each sampled household using principal component analysis and the households are then subdivided into equal-size wealth quintiles. Distribution of wealth is uneven across different contexts, with the highest (wealthiest) SES quintile households under-represented in rural contexts. In rural Tanzania (DHS 2015–16), there were only 14.6% and 2.5% women in Quintile 4 (richer) and Quintile 5 (richest), respectively. Thus, for the purpose of this analysis, the two highest wealth quintiles were merged, resulting in the creation of four wealth groups (poorest, poorer, middle and wealthiest). The terms richest/wealthiest refer to relative wealth in a poor, rural context, thus indicate women from the least poor households. To analyse the interaction of SES and parity, a binary SES variable was created, by generating two equal groups based on wealth scores (wealth score < median recoded as poorer, wealth score ≥ median coded as richer 50%).

*Parity group* refers to a woman’s parity at index pregnancy (0, 1–2, 3–4 and ≥5). Grand multiparity was defined as parity ≥5 (Mgaya *et al.*, 2013).

*Maternal age at index birth* was coded in 5-year age groups, grouping categories at the extremes of age because they had fewer than 100 observations (≤19, 20–24, 25–29, 30–34, 35–39 and ≥40 years). The 20–24 years’ group was used as reference.

*Maternal education* was recoded into three categories: no education, completed primary and completed secondary or higher.

*Marital status* at survey was recoded into currently married/cohabiting and not currently married/cohabiting.

*Zone of residence:* Tanzania is divided into 21 administrative regions, grouped into eight zones (Tanzania MoHCDGEC, MoH [Zanzibar], and ICF, 2016). We used the eight zones to account for sub-national variation in outcomes and service availability (Armstrong *et al.*, 2016). All eight zones include rural areas; the Eastern zone includes the Dar es Salaam urban conglomerate.

*Antenatal care (ANC) for the index pregnancy* was categorized into no visits, 1–3 visits and ≥4 visits.

Other obstetric characteristics studied were the following: multiple index birth, a previous birth in the recall period by Caeserean section (CS), death of a previous child (born in the recall period) aged 1–12 months, death of a previous child (born in the recall period) aged <1 month, a short preceding birth interval (≤12 months).

### Statistical analysis

Analysis was performed using STATA IC 15 software. Complex survey design and non-response (stratification, clustering and survey weights) were accounted for using svyset commands. Characteristics of the sample were analysed with proportions and 95% confidence intervals (CIs) of outcome and exposure variables. There were no missing data for the variables examined. Proportions of subgroups of women at each level of outcome (hospital/PHC/home birth) for each exposure variable were examined using bivariate analysis. As the interaction between SES and parity was of interest, the proportion of women giving birth at each location by combinations of parity levels and a binary SES variable (poorer/richer) were determined. Associations between the outcome variable and dependent variables (demographic, geographical characteristics, SES, ANC care received and available obstetric factors) were assessed in bivariate analysis. Variables which were significant at *P* <0.05 level in bivariate analysis were included in the final multivariable model. Multinomial logistic regression was used as the outcome variable had three categories, thus allowing to include all births in one model. The baseline outcome was birth in PHC, thus the model produced odds ratios (ORs) of home vs PHC and hospital vs PHC birth. In the final multivariable model, we tested for an interaction between SES and parity group. We calculated the margins to obtain predicted percentages of women giving birth at each of the three locations, depending on their SES and parity group combination. Results were used to calculate the difference or gap in hospital or PHC uptake for birth between the wealthiest and poorest women.

### Ethical approval

The DHS receive government permission, use informed consent and assure respondents of confidentiality. Permission to use the dataset for the purpose of this analysis was obtained from the DHS programme.

## Results

### Population characteristics

Observations of 4456 women living in rural mainland Tanzania and the circumstances of their most recent live birth in the 5 years preceding the survey were included in the analysis. Home birth was reported by 41% of women and a slight majority reported a facility birth (59%): 35% in PHCs and 24% in hospitals. Women from the wealthiest households were under-represented, with only 17% in the highest group compared with 28% in the lowest. Approximately one in five women was nulliparous at index birth (22%), while 25% had parity five or higher. Background characteristics are summarized in Supplementary Table S1.

Results of bivariate analysis are reported in Table 1. The percentage of rural women using hospitals for childbirth increased with higher SES, from 16% in the poorest group to 45% in the wealthiest. PHC births also rose with increasing wealth, although less steeply than hospital births. As parity increased, hospital births reduced sharply, while PHC births had a less clear trend across SES and parity and varied only marginally at around one-third of births. Hospital births increased with higher maternal age, maternal education and number of ANC visits. There was a wide variation in hospital births across the eight zones, ranging from 16% in the Lake Zone to 39% in the Southern Highlands. PHC facilities provided a substantial proportion of childbirth care in rural areas of all zones (range 23–48%, median 38%).

**Table 1.** Percentages of home, primary care facility or hospital birth by subgroups among rural women with a recent live birth (TDHS 2015–16) *n* = 4456

			Home births		PHC facility births		Hospital births	
Variable		Women in category (%)	**Number**	**% (95% CI)**	**Number**	**% (95% CI)**	**Number**	**% (95% CI)**
SES								
Poorest		1351 (29.3)	742	55.5 (50.5–60.3)	413	29.1 (25.4–33.0)	196	15.5 (12.3–19.1)
Poorer		1239 (28.4)	559	45.7 (41.5–50.0)	467	36.5 (32.7–40.5)	213	17.8 (14.9–21.1)
Medium		1104 (25.2)	383	35.5 (31.1–40.1)	448	39.7 (35.5–44.0)	273	24.9 (21.5–28.6)
Wealthiest		762 (17.1)	131	18.1 (14.1–23.0)	288	36.9 (31.9–42.2)	343	45.0 (39.4–50.7)
Parity								
0		963 (22.2)	245	25.6 (22.2–29.4)	333	32.9 (29.2–36.8)	385	41.5 (37.1–46.1)
1–2		1342 (30.6)	537	40.6 (36.4–45.1)	514	37.3 (33.5–41.3)	291	22.1 (19.0–25.5)
3–4		1057 (23.6)	475	45.4 (41.1–50.0)	410	38.9 (35.0–42.9)	172	15.7 (12.9–18.9)
≥5		1094 (23.6)	558	52.7 (48.2–57.2)	359	30.9 (27.5–34.6)	177	16.4 (13.6–19.6)
Maternal age at index birth (years)								
≤19		743 (17.6)	241	32.0 (27.7–36.6)	280	36.2 (32.0–40.7)	222	31.8 (27.2–36.8)
20–24		1124 (25.0)	459	41.6 (37.7–45.7)	412	35.7 (32.1–39.4)	253	22.7 (19.6–26.1)
25–29		946 (21.2)	400	42.9 (38.2–47.8)	343	35.7 (31.6–40.0)	203	21.4 (18.0–25.2)
30–34		744 (16.3)	321	43.9 (38.7–49.3)	255	33.4 (28.9–38.1)	168	22.7 (18.9–27.1)
35–39		594 (13.1)	249	43.7 (38.2–49.4)	228	35.7 (30.8–41.0)	117	20.6 (16.4–25.4)
40–49		305 (6.8)	145	47.9 (41.4–54.5)	98	32.3 (26.8–38.4)	62	19.7 (15.2–25.3)
Maternal education at survey								
No education		1054 (23.8)	577	54.7 (49.8–59.5)	343	32.5 (28.7–36.7)	134	12.8 (10.2–15.9)
Completed primary		3028 (67.5)	1191	40.2 (37.0–43.5)	1120	35.6 (32.9–38.5)	717	24.2 (21.6–27.0)
Secondary and above		374 (8.7)	47	13.1 (9.6–17.7)	153	38.9 (33.1–45.0)	174	48.0 (41.7–54.4)
Marital status at survey								
Currently married or cohabiting		3695 (82.9)	1548	43.6 (39.1–46.1)	1334	35.0 (32.4–37.8)	813	22.4 (20.0–25.1)
Not currently married or cohabiting		761 (17.1)	267	35.1 (30.5–40.0)	282	35.9 (31.6–40.5)	212	29.0 (24.8–33.6)
Zone of residence								
Western		515 (14.0)	255	50.1 (39.4–60.9)	170	31.8 (24.3–40.4)	90	18.1 (11.8–26.6)
Northern		420 (10.1)	164	40.7 (28.7–54.0)	95	22.6 (16.8–29.8)	161	36.6 (26.1–48.6)
Central		569 (14.0)	246	41.6 (32.6–51.2)	165	31.1 (24.4–38.8)	158	27.3 (20.9–34.8)
Southern Highlands		394 (6.0)	48	14.3 (7.2–26.6)	189	46.3 (34.9–58.0)	157	39.4 (30.1–49.5)
Southern		273 (5.5)	53	19.5 (12.5–29.0)	128	47.6 (39.5–55.8)	92	33.0 (25.3–41.7)
Southern West Highlands		588 (11.0)	256	37.5 (27.9–48.1)	255	43.5 (34.1–53.3)	77	19.1 (12.9–27.3)
Lake		1440 (32.3)	732	51.2 (46.4–55.9)	487	32.6 (28.8–36.5)	221	16.3 (13.0–20.3)
Eastern		257 (7.1)	61	24.6 (15.7–36.5)	127	47.8 (35.9–60.0)	69	27.6 (19.1–38.2)
Antenatal visits								
None		99 (2.3)	78	77.7 (68.3–84.9)	14	13.9 (7.8–23.5)	7	8.5 (3.9–17.5)
1–3		2344 (52.4)	1088	46.9 (42.9–51.0)	783	32.4 (29.2–35.8)	473	20.7 (18.1–23.5)
≥4		2013 (45.4)	649	33.0 (29.7–36.4)	819	39.4 (36.5–42.4)	545	27.6 (24.7–30.8)
Index pregnancy was multiple								
No		4371 (98.0)	1783	41.3 (38.1–44.6)	1591	35.4 (32.8–38.0)	997	23.4 (20.9–26.0)
Yes		85 (2.0)	32	40.6 (29.4–53.0)	25	26.4 (17.6–37.5)	28	33.0 (22.8–45.1)
Previous birth was by CS								
No or no previous birth		4428 (99.1)	1811	41.5 (38.2–44.8)	1612	35.3 (32.7–37.9)	1005	23.3 (20.9–25.9)
Yes		28 (0.6)	4	13.9 (5.1–32.9)	4	17.0 (6.1–39.5)	20	69.1 (47.9–84.4)
Short previous birth interval (≤12 months)								
No or no previous birth		4410 (99.1)	1791	41.2 (38.0–44.5)	1601	35.2 (32.6–37.8)	1018	23.6 (21.2–26.2)
Yes		46 (0.9)	24	51.6 (36.0–66.9)	15	33.1 (19.5–50.3)	7	15.3 (7.2–29.7)
Death of newborn preceding index birth								
No or no previous birth		4377 (98.0)	1783	41.3 (38.1–44.6)	1586	35.1 (32.6–37.8)	1008	23.6 (21.2–26.2)
Yes		79 (2.0)	32	38.5 (27.4–51.0)	30	38.1 (26.7–51.0)	17	23.4 (14.2–36.0)
Death of child born before index birth aged >1 month and <12 months								
No		4372 (98.0)	1771	41.0 (37.8–44.4)	1589	35.3 (32.7–37.9)	1012	23.7 (21.3–26.3)
Yes		84 (2.0)	44	53.3 (41.1–65.1)	27	31.1 (21.2–43.1)	13	15.6 (8.6–26.7)
Parity by SES (*n* = 4456)								
Parity 0 (*n* = 963)	Poorer 50%	412 (43.5)	145	34.2 (28.4–40.6)	142	32.6 (27.3–38.4)	125	33.2 (26.5–40.7)
	Richer 50%	551 (56.5)	100	19.0 (15.1–23.6)	191	33.1 (28.2–38.4)	260	47.9 (42.5–53.4)
Parity 1–2 (*n* = 1342)	Poorer 50%	647 (46.5)	337	53.7 (48.4–59.0)	238	35.5 (30.8–40.6)	72	10.7 (8.1–14.1)
	Richer 50%	695 (53.5)	200	29.2 (24.3–34.7)	276	38.8 (33.7–44.2)	219	32.0 (27.5–36.8)
Parity 3–4 (*n* = 1057)	Poorer 50%	533 (49.5)	303	58.7 (53.2–64.0)	170	31.4 (27.0–36.4)	60	10.0 (7.4–13.3)
	Richer 50%	524 (50.5)	172	32.5 (27.3–38.1)	240	46.3 (40.7–52.0)	112	21.3 (17.1–26.1)
Parity ≥5 (*n* = 1094)	Poorer 50%	636 (57.8)	366	59.1 (53.6–64.4)	187	27.4 (23.1–32.0)	83	13.6 (10.5–17.4)
	Richer 50%	458 (42.2)	192	44.0 (38.4–49.9)	172	35.8 (30.9–41.0)	94	20.2 (16.3–24.8)

Examining SES and parity together, hospital births were more frequent in women from wealthier households in all parity groups, with percentages reducing as parity increased across all wealth groups. The drop of hospital use for childbirth was seen among poorer women already at Parity 1–2, while among wealthier women, this reduction was seen at Parity 3–4. Despite the decrease, the percentage of births in hospitals remained higher among wealthier than poorer women in all parity groups. The gap between the poorest and wealthiest women in hospital births was greatest at Parity 1–2 (Supplementary Graph 1).

### Logistic regression

Results of bivariate and multivariate logistic regression are reported in Table 2.

**Table 2.** Crude and adjusted ORs by multinomial logistic regression of home vs PHC birth (left) and hospital vs primary care births (right) in rural women, with a live birth in the last 5 years (Tanzania, DHS 2015–16)

	Home birth vs PHC birth				Hospital birth vs PHC birth			
Variable	Crude OR	*P*-value	Adjusted OR^a^	*P*-value	Crude OR	*P*-value	Adjusted OR^a^	*P*-value
SES								
Poorest	ref		ref		ref		ref	
Poorer	0.66 (0.51–0.84)	1	0.75 (0.58–0.96)	23	0.92 (0.67–1.26)	0.6	0.90 (0.66–1.23)	501
Medium	0.47 (0.36–0.61)	<0.001	0.56 (0.43–0.74)	<0.001	1.18 (0.86–1.62)	0.3	1.05 (0.78–1.41)	731
Wealthiest	0.26 (0.18–0.37)	<0.001	0.34 (0.23–0.50)	<0.001	2.30 (1.61–3.27)	<0.001	1.78 (1.26–2.50)	0.001
Parity								
0	0.71 (0.56–0.91)	0.006	0.84 (0.63–1.12)	0.225	2.13 (1.67–2.73)	<0.001	3.22 (2.34–4.43)	<0.001
1–2	ref		ref		ref		ref	
3–4	1.07 (0.87–1.31)	0.5	1.07 (0.82–1.39)	0.642	0.68 (0.51–0.90)	0.007	0.46 (0.32–0.65)	<0.001
≥5	1.57 (1.26–1.95)	<0.001	1.54 (1.05–2.25)	0.028	0.89 (0.67–1.19)	0.4	0.59 (0.39–0.89)	0.013
Maternal age at birth								
≤19	0.76 (0.60–0.96)	0.02	0.80 (0.59–1.08)	0.151	1.38 (1.05–1.80)	0.019	0.86 (0.62–1.19)	0.366
20–24	ref		ref		ref		ref	
25–29	1.03 (0.82–1.29)	0.796	0.86 (0.66–1.13)	0.289	0.94 (0.72–1.24)	0.664	1.89 (1.37–2.60)	<0.001
30–34	1.13 (0.89–1.43)	0.322	0.80 (0.56–1.13)	0.204	1.07 (0.81–1.42)	0.636	2.69 (1.78–4.08)	<0.001
35–39	1.05 (0.81–1.35)	0.724	0.61 (0.40–0.93)	0.021	0.90 (0.64–1.27)	0.551	2.49 (1.51–4.13)	<0.001
40–49	1.27 (0.91–1.76)	0.156	0.69 (0.44–1.08)	0.104	0.96 (0.65–1.41)	0.829	2.64 (1.58–4.42)	<0.001
Maternal education at survey								
No education	1.49 (1.23–1.80)	<0.001	1.19 (0.97–1.45)	0.094	0.58 (0.44–0.75)	<0.001	0.70 (0.53–0.93)	0.013
Primary	ref		ref		ref		ref	
Secondary and above	0.30 (0.21–0.43)	<0.001	0.38 (0.25–0.59)	<0.001	1.82 (1.39–2.39)	<0.001	1.03 (0.76–1.41)	0.837
Marital status at survey								
Currently married or cohabiting	ref				ref			
Not currently married or cohabiting	0.80 (0.64–1.01)	0.059			1.26 (1.00–1.59)	0.049		
Zone of residence								
Western	1.00 (0.63–1.60)	0.989	0.82 (0.51–1.31)	0.401	1.13 (0.66–1.93)	0.645	1.28 (0.73–2.23)	0.394
Northern	1.15 (0.67–1.96)	0.621	1.40 (0.86–2.28)	0.181	3.23 (1.95–5.34)	<0.001	2.54 (1.56–4.13)	<0.001
Central	0.85 (0.54–1.34)	0.483	0.89 (0.57–1.39)	0.61	1.75 (1.12–2.74)	0.014	1.83 (1.16–2.88)	0.01
Southern Highlands	0.20 (0.09–0.46)	<0.001	0.24 (0.11–0.57)	0.001	1.70 (1.01–2.86)	0.045	1.52 (0.90–2.29)	0.117
Southern	0.26 (0.15–0.45)	<0.001	0.27 (0.16–0.47)	<0.001	1.39 (0.89–2.16)	0.151	1.44 (0.90–2.29)	0.124
Southern West Highlands	0.55 (0.34–0.90)	0.016	0.55 (0.34–0.91)	0.019	0.88 (0.51–1.52)	0.637	0.85 (0.49–1.49)	0.579
Lake	ref		ref		ref		ref	
Eastern	0.33 (0.18–0.61)	0.001	0.43 (0.22–0.83)	0.012	1.15 (0.65–2.06)	0.63	1.17 (0.67–2.07)	0.58
ANC (visits)								
None	6.71 (3.53–12.74)	<0.001	5.75 (3.23–10.23)	<0.001	0.87 (0.29–2.58)	0.801	0.87 (0.25–3.07)	0.828
1–3	1.73 (1.45–2.07)	<0.001	1.53 (1.28–1.83)	<0.001	0.91 (0.76–1.08)	0.276	1.10 (0.92–1.32)	0.305
≥4	ref		ref		ref		ref	
Multiple live birth at index pregnancy								
No	ref		ref		ref		ref	
Yes	1.32 (0.75–2.31)	0.332	1.17 (0.68–2.00)	0.578	1.89 (1.02–3.50)	0.042	2.24 (1.18–4.26)	0.014
Previous birth was by CS								
No	ref		ref		ref		ref	
Yes	0.69 (0.16–3.03)	0.628	0.71 (0.18–2.84)	0.626	6.15 (1.89–20.0)	0.003	6.96 (2.15–22.57)	0.001
Short preceding birth interval (≤12 months)								
No	ref				ref			
Yes	1.33 (0.63–2.79)	0.45			0.69 (0.26–1.84)	0.457		
Previous neonatal death								
No	ref				ref			
Yes	0.86 (0.49–1.51)	0.596			0.92 (0.46–1.83)	0.803		
Previous baby died								
No	ref				ref			
Yes	1.47 (0.85–2.56)	0.169			0.75 (0.35–1.58)	0.441		

a Adjusted for wealth, parity, maternal age at index birth, maternal education, marital status, ANC visits and multiple index pregnancy.

In adjusted analysis, compared with women from the poorest households’ group, all wealthier women were less likely to have given birth at home vs in PHC. The wealthiest were 66% less likely to do so. High-parity women (≥5) had higher odds of home birth (OR 1.54, 95% CI 1.05–2.25) compared with the reference group Parity 1–2, while odds in other parity groups were not significantly different from baseline. Higher odds of a home birth were seen in women with no ANC or 1–3 ANC visits compared with women with ≥4 visits. Compared with women with primary education, those with no education had higher odds of a home birth, while those with secondary or higher education had reduced odds. Women residing in four zones (Southern Highlands; Southern and Southern West Highlands; and Eastern) had reduced odds compared with those residing in the reference Lake Zone.

In adjusted analysis, the wealthiest rural women had higher odds (OR 1.78, 95% CI 1.26–2.50) of a hospital vs a PHC birth compared with the poorest, while other wealth groups were not significantly different from the poorest. Higher odds of hospital vs PHC birth were found in Parity 0 women compared with baseline Parity 1–2 (OR 3.22, 95%CI, 2.34–4.43), while the odds were reduced in higher-parity groups. The effect of maternal age was confounded in crude analysis; in adjusted analysis, the odds of hospital vs PHC birth increased with age. Women with a previous birth by CS had higher odds of a hospital birth compared with those with no previous CS, while women with no education, compared with those with primary education, had reduced odds of hospital vs PHC birth. Higher odds were observed in women residing in two zones (Northern and Central) compared with those residing in the reference Lake Zone.

### Interaction between SES and parity

To assess the joint effects of parity and SES, the final adjusted multinomial logistic regression model was run with an interaction term between the two variables. A likelihood ratio test comparing the model with and without interaction indicated better fit of the model with interaction (*P* = 0.006). The reference group included the poorest women at parity ≥5, as this group had the lowest use of hospital-based childbirth care and was the most numerous wealth/parity subgroup (*n* = 406). Results are shown in Supplementary Tables S2a and S2b.

All combinations of SES and parity had lower odds than the baseline category of a home vs a PHC birth, although not all reached statistical significance at *P* < 0.05. The richest women at high parity (≥5) had the lowest adjusted OR (0.29, 95% CI 0.15–0.57) compared with the reference group. The poorest women at Parity 0 had an OR of 8.03 (95% CI, 4.45–14.46) compared with the baseline of a hospital vs PHC birth, while at other parity levels the ORs were not significantly different. Women at Parity 1–2 from poorer, medium and richest groups had higher odds of hospital vs PHC childbirth compared with the baseline group; the OR was non-significant in the poorest group. In other groups, ORs were not significantly different from the baseline.

**Graph 1.**
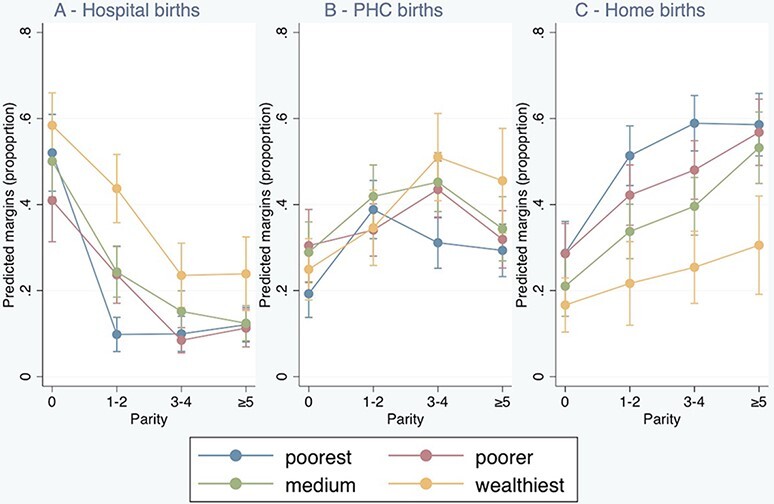
Predicted margins of birth at each location, rural Tanzania 2015-16, 95% CI

We predicted the percentages of childbirth for each combination of SES and parity in each location using margins analysis; results are reported in Table 3 and displayed in Graph 1(A-C). Across all SES groups, hospital-based childbirth (Graph 1A) was highest at first birth, at >40%. Use of hospitals reduced in all SES groups with increasing parity, but the shape of this decline varied. Among the wealthiest women, hospital use decreased gradually, reaching its lowest (around 25%) at parity 3–4. Among the poorest, the decline was abrupt after parity 0, levelling out at 12% at parity 1–2. The effect of wealth on PHC births was more complex (Graph 1B). The predicted percentages at this level were lowest among nulliparous women in all wealth groups. Among the wealthiest, the percentage rose with parity, reaching its maximum (51%) at parity 3–4. Among the poorest women, the predicted utilization reached the highest level at parity 1–2 (39%) and then levelled off at around 30%. In all wealth groups, after parity 0, ≥30% of women were predicted to give birth in a PHC. Median utilization of PHC facilities in parous women was 35% (range 30–51%), while in women at first parity it was 27% (range 20–31%). The percentage of births at home (Graph 1C) increased as parity rose in all wealth groups and was lowest among the wealthiest women in all parity groups.

**Table 3.** Predicted margins (%) for each outcome (home/PHC/hospital birth) in rural women, Tanzania 2015–16 DHS, by SES and parity, adjusted for wealth, parity, maternal age at index birth, maternal education, marital status, ANC visits and multiple index pregnancy

Predicted margins (%) of rural women by outcome, SES group and parity					
Outcome	SES group	Parity 0	Parity 1–2	Parity 3–4	Parity ≥5
Home birth	Poorest	28 (21–35)	51 (44–58)	59 (52–65)	58 (51–65)
	Poorer	28 (21–35)	42 (35–49)	47 (40–54)	56 (48–63)
	Medium	21 (14–28)	33 (27–39)	39 (33–46)	53 (45–61)
	Wealthiest	16 (10–22)	21 (11–30)	25 (17–33)	30 (19–41)
PHC birth	Poorest	20 (14–25)	39 (33–46)	31 (25–37)	30 (24–36)
	Poorer	31 (22–39)	34 (28–40)	44 (38–51)	32 (26–39)
	Medium	28 (22–35)	42 (35–49)	46 (39–52)	35 (27–42)
	Wealthiest	25 (18–32)	35 (26–43)	51 (41–61)	47 (35–59)
Hospital birth	Poorest	52 (43–61)	10 (6–14)	10 (6–14)	12 (8–16)
	Poorer	42 (32–51)	23 (17–30)	8 (6–11)	12 (8–16)
	Medium	51 (43–59)	25 (19–30)	15 (10–20)	12 (8–16)
	Wealthiest	59 (51–66)	44 (37–52)	25 (17–32)	23 (15–31)

**Graph 2.**
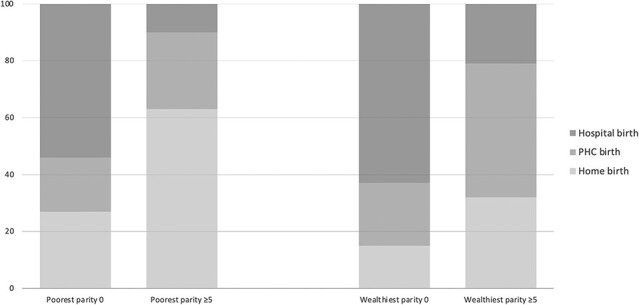
Predicted margins (percentages) of Hospital/PHC/Home births of poorest and wealthiest rural women by parity, Tanzania 2015-16

The profiles of birth location among the two extremes of wealth (poorest and richest women) are compared in Graph 2. Among the richest women, there was a shift in the location of births from mainly hospital (at parity 0) to mainly PHC facilities (at parity ≥5). In the poorest women’s group, between parity groups 0 and ≥5, decline in hospital births was accompanied by a sharp rise in home births, with a small increase in PHC births.

## Discussion

This study explored rural women’s differential use of childbirth care in Tanzania. We report three key findings. First, there was a socio-economic inequity in rural women’s use of hospital-based childbirth, which additionally varied with parity. Second, the poorest multiparous women had the lowest use of hospital-based care for childbirth (around 10%), with no increase in uptake at grand multiparity despite increased risk. This group also had lower uptake of PHC care. Third, PHC facilities provided care to a sizeable proportion of women after the first birth, with a median uptake of 35% (range 30–51%) by women after the first birth (compared with a median of 27% by women at first birth).

The poorest women in rural Tanzania were less likely than those from the wealthiest households to give birth in hospitals, where advanced management of childbirth complications was available. The study adds to existing evidence that wealth is not just a determinant for facility birth, but also for uptake of hospital-based childbirth care within the health system. It expands findings of a previous sub-national study (Straneo *et al.*, 2014) and earlier studies (Benova *et al.*, 2014). We found that the gap between the poorest and wealthiest women in use of hospitals was less pronounced among nulliparous women. Health policy in Tanzania (Tanzania MoHSW, 2008) recommends that women’s first births should take place in hospitals. In spite of the existing recommendations, uptake by rural women at first birth was not universal, as just over half used hospital-based care. We found that hospital-based care use among nulliparous women was very similar across wealth groups. Factors other than wealth are likely to limit hospital use at first birth; amongst these distance to hospital from a woman’s residence, which could not be accounted for, stands out, and interaction between distance and wealth has been described (Bai *et al.*, 2002; Hanson *et al.*, 2015; Wong *et al.*, 2020). Our finding is in line with that of other researchers indicating that women and their families recognize the first birth as a higher-risk one (Jahn *et al.*, 1998; Dunlop *et al.*, 2018). Utilization of hospitals decreased at different rates in SES groups with increasing parity and the gap between the poorest and wealthiest was widest at Parity Level 1–2 but persisted across all higher-parity groups. A switch in birth location away from facilities between first- and second-order births was found to be less likely in wealthier households across low- and middle-income countries (Benova *et al.*, 2017). What this study adds is that among the poorest women between first-order and successive births (Parity 1–2), there was a switch within the health system, from hospital-based care to PHC-based care, and to home-based care.

Utilization of hospital-based childbirth is lowest among the poorest, multiparous women. Use, as estimated by margins analysis, dropped to around 10% at all levels of parity after the first-order birth. Despite greater risk in women with ≥5 previous births of adverse pregnancy outcomes, including haemorrhagic complications (Bai *et al.*, 2002; Mgaya *et al.*, 2013; Filippi *et al.*, 2016), there was no increased hospital care uptake among the poorest women. Factors contributing to this may be inadequate counselling during ANC on hospital-based childbirth resulting in low perceived risk (Pembe *et al.*, 2008), childcare duties at home, and greater economic constraints due to larger families. Reducing facility use with increasing parity is well documented (Kyei-Nimakoh *et al.*, 2017; Moyer and Mustafa, 2013; Gabrysch and Campbell, 2009; Berhan and Berhan, 2014). Results of our study add that, in rural Tanzania, multiparous women will opt for a home birth when economic means are limited but will uptake PHC-based childbirth when resources are available. Poor multiparous women constitute a disadvantaged group, least served by hospitals or indeed by any facility. Qualitative studies in southern Tanzania (Kowalewski *et al.*, 2000) indicated that in the community, these women were perceived as vulnerable due ‘to fatigue and (being) overburdened with household duties’, and precisely these factors prevented them from accessing health services.

Although our analysis focused on hospital births, rural women’s uptake of childbirth care in lower-level facilities is noteworthy. PHC units (health centres and dispensaries) have a critical role in childbirth care in rural Tanzania, as 61% of all facility births took place here, and utilization ranged from 30 to 51% (median 35%) across all SES groups after Parity 0. The greatest use is among the wealthiest, at intermediate and high parity. This has relevance in the current debate on reorganization of maternity care in low-income countries (Campbell *et al.*, 2016; Kruk *et al.*, 2018; Hanson and Schellenberg, 2019). From these data, childbirth care in PHC facilities is used by all wealth groups: among the wealthiest, uptake increases with parity, while among the poorest, utilization is mostly at intermediate levels of parity. Comparing the shift in births between the two extremes of parity in the poorest and richest women suggests that, in this context, a reduction in the availability of facilities providing childbirth care without other measures may result in an increase in the already-high level of home births among the poorest women.

Policy recommendations arising from this study include three main points. Firstly, aiming attention on the poorest women allows identification of health system adjustments to mitigate the effects of poverty on childbirth-related deaths (WHO, 2008). A subsidized voucher scheme has been applied in Kenya (Dennis *et al.*, 2019; Wong *et al.*, 2020). Maternity waiting homes may contribute to facilitating access to hospitals (Virgo *et al.*, 2017), and there is evidence that they are utilized preferentially by poorer women (Fogliati *et al.*, 2017). Secondly, high-parity women’s low use of hospital-based childbirth care, particularly among the poorest, requires urgent action. All women should receive appropriate, timely care. National policy should focus attention on grand-multiparous women as a particularly higher-risk group. Guidelines should be in place to prepare these women for hospital-based births. They may include adapted birth preparedness plans and emergency transport during labour to improve geographic accessibility. Thirdly, the current debate on centralization of childbirth care must take into account the sizeable proportions of women using PHC facilities for obstetric care. Care at childbirth is part of essential care, as defined in the Alma Ata declaration of PHC (Beard and Redmond, 1979), which also includes the ‘scientifically sound’ concept. Effective coverage is increasingly advocated, in place of contacts with care (Campbell *et al.*, 2016; Marsh *et al.*, 2020). In this context, to achieve effective coverage for the large proportion of women who uptake PHC-based care, quality-adjusted coverage (Marsh *et al.*, 2020) must be available at the base of the health system pyramid (Hanson and Schellenberg, 2019; Straneo *et al.*, 2014; Fogliati *et al.*, 2015). Comprehensive EmOC in strategically identified rural health centres is one possible solution (Nyamtema *et al.*, 2016); a locally adapted and community-participated reduction of birth sites may be necessary to balance quality and coverage of care (Fogliati *et al.*, 2015). From a policy perspective, the position of childbirth care in PHC should be reappraised.

### Limitations

This analysis is the first, to the best of our knowledge, to examine the use of different levels of the health system for childbirth among rural women in Tanzania and analysed the interaction between wealth and parity. It is based on nationally representative data, from a country that has consistently supported the development of a PHC network (Tanzania MoHSW, 2007; Tanzania MoHSW, MoHMZ, NBS, OCGS, and ICF, 2015), and thus is a model for countries developing rural obstetric care. The Tanzania DHS is unique in allowing identification of facility type (hospital, health centre or dispensary) in both the public and the private sectors, thus providing a more detailed picture of where women report giving birth (Tanzania MoHCDGEC, 2016). The DHS data set was complete, with very little non-response and missing data. Multinomial logistic regression allowed us to include all three locations in one model and thus study factors significant in use of hospital vs PHC facilities, and home vs PHC. Some caution should be applied when interpreting the findings, in terms of the cross-sectional nature of the DHS data and the possible response bias. Since in Swahili all facilities may be referred to as ‘hospitali’, lower-level public health facilities may be misreported as district hospitals. The DHS interviewers are instructed to circle a type of facility, if known, and if not, to write down the name of the facility, which is later coded as a specific type of facility by the field supervisor. This non-differential misclassification of facilities may bias results and may have led to weaker associations. This DHS collects limited information on obstetric risk factors (Virgo *et al.*, 2017); thus, use of facilities due to risk factors identified during ANC (such as hypertension or maternal infections) cannot be fully captured. Even the risk factors for which some information is available may not fully reflect women’s knowledge prior to index birth; e.g., limitations in DHS questionnaire identification of twin pregnancies have been described recently (Hanson *et al.*, 2019). This is unlikely to modify findings, as in a study on four East African countries no association between obstetric risk and birth location was found (Virgo *et al.*, 2017), while wealth and education were strong determinants. Information on referral was lacking; thus, hospital births may include women who had been referred during labour from a PHC unit. In previous studies we found that intra-partum referral rates were very low (Straneo *et al.*, 2016; Fogliati *et al.*, 2017); thus, this too is unlikely to change the findings. Distance travelled to facilities could not be taken into account. Recent studies indicate that travel time is an important factor for hospital births in Tanzania (Wong *et al.*, 2020), and current distribution leaves the rural poor underserved (Wong *et al.*, 2019). Additionally, the study broadly categorized facilities by level (PHC and hospitals) but could not account for variation of quality of care within levels, such as more limited quality at hospital level or more advanced care in health centres (Nyamtema *et al.*, 2016; Tanzania MoHSW, MoHMZ, NBS, OCGS, and ICF, 2015).

In conclusion, the study found that in rural Tanzania the use of hospital-based childbirth was not equitable. Inequity varied with parity level: at first birth, uptake varied only minimally with wealth, while in successive births it was strongly dependent on SES and parity. Uptake was the lowest amongst the poorest, multiparous women, with no increase in uptake at grand multiparity (≥5), in spite of increased risk. PHC-based childbirth accounted for a median of 35% of births after the first in this setting.

To leave no one behind in attaining Sustainable Development Goal 3 on maternal and preventable newborn mortality (Boerma *et al.*, 2018), it is necessary to identify who is underserved at childbirth and make adjustments to improve the use of high-quality care, bearing in mind that from a human rights’ perspective health care should contribute to equity (Tudor Hart, 1971). Strategies are needed to improve uptake of hospital-based care among the poorest, rural women, particularly at high parity. A reassessment of the whole district health system, which may involve re-evaluation of childbirth care in PHC and strengthening referral linkages, would benefit a substantial proportion of women.

## Supplementary Material

czab079_Supp

## Data Availability

The dataset is available to the public at www.measuredhs.com.
