# Supplementary material for: Inequity in uptake of hospital-based childbirth care in rural Tanzania: analysis of the 2015–16 Tanzania Demographic and Health Survey
Source: Health Policy Plan. 2021 Jul 19;36(9):1428–40. doi: 10.1093/heapol/czab079 (PMC8505858; doi:10.1093/heapol/czab079)
Supplement: czab079_Supp [file czab079_supp.zip › Supplementary_Table_1.docx]

*Supplementary Table 1 - Characteristic of rural women with a recent birth from Tanzania DHS 2015-16 (n=4456)*

| Variables/categories (n=4456) |  | N | % (95% CI) |
| --- | --- | --- | --- |
| Birth location | |  |  |
| Home |  | 1815 | 41.3 (38.1-44.6) |
| Primary health care facility |  | 1616 | 35.2 (32.6-37.8) |
| Hospital |  | 1025 | 23.5 (21.1-26.1) |
| Socio-economic status | |  |  |
| Poorest |  | 1351 | 29.3 (26.3-32.5) |
| Poorer |  | 1239 | 28.4 (26.4-30.4) |
| Medium |  | 1104 | 25.2 (23.1-27.4) |
| Wealthiest |  | 762 | 17.1 (14.7-19.8) |
| Parity at index birth | |  |  |
| 0 |  | 963 | 22.2 (20.8-23.8) |
| 1-2 |  | 1342 | 30.6 (28.9-32.3) |
| 3-4 |  | 1057 | 23.6 (22.2-25.2) |
| ≥5 |  | 1094 | 23.6 (22.0-25.2) |
| Maternal age at index birth | |  |  |
| ≤19 |  | 743 | 17.6 (16.3-19.0) |
| 20-24 |  | 1124 | 25.0 (23.6-26.5) |
| 25-29 |  | 946 | 21.2 (20.0-22.6) |
| 30-34 |  | 744 | 16.3 (15.0-17.6) |
| 35-39 |  | 594 | 13.1 (12.1-14.2) |
| 40-49 |  | 305 | 6.8 (6.0-7.7) |
| Maternal education at survey | |  |  |
| No education |  | 1054 | 23.8 (21.6-26.1) |
| Completed primary |  | 3028 | 67.5 (65.3-69.6) |
| Secondary and above |  | 374 | 8.7 (7.6-10.0) |
| Marital status at survey | |  |  |
| Currently married or cohabiting |  | 3695 | 82.9 (81.4-84.3) |
| Not currently married or cohabiting |  | 761 | 17.1 (15.7-18.6) |
| Zone of residence | |  |  |
| Western | | 515 | 14.0 (11.8-16.5) |
| Northern | | 420 | 10.1 (8.9-11.4) |
| Central | | 569 | 14.0 (12.4-15.8) |
| Southern Highlands | | 394 | 6.0 (5.3-6.8) |
| Southern | | 273 | 5.5 (4.8-6.2) |
| South West Highlands | | 588 | 11.0 (9.1-13.2) |
| Lake | | 1440 | 32.3 (30.0-34.7) |
| Eastern | | 257 | 7.1 (6.0-8.5) |
| Antenatal visits during index pregnancy | |  |  |
| None |  | 99 | 2.3 (1.7-2.9) |
| 1-3 |  | 2344 | 52.4 (50.1-54.6) |
| ≥4 |  | 2013 | 45.4 (43.1-47.7) |
| Multiple live index birth | |  |  |
| No |  | 4371 | 98.0 (97.5-98.4) |
| Yes |  | 85 | 2.0 (1.6-2.5) |
| Previous birth in recall period by Caesarean section | |  |  |
| No or no previous birth |  | 4428 | 99.4 (99.1-99.6) |
| Yes |  | 28 | 0.6 (0.4-0.9) |
| Short previous birth interval (≤12 months) | |  |  |
| No or no previous birth |  | 4410 | 99.1 (98.7-99.3) |
| Yes |  | 46 | 0.9 (0.7-1.3) |
| Death of new-born preceding index birth | |  |  |
| No or no previous birth |  | 4377 | 98.4 (98.0-98.8) |
| Yes |  | 79 | 1.6 (1.2-2.0) |
| Death of child born before index birth aged 1-12 months | |  |  |
| No or no previous birth |  | 4372 | 98.0 (97.5-98.4) |
| Yes |  | 84 | 2.0 (1.6-2.5) |
| Parity by SES | |  |  |
| Parity 0 (n=963) | Poorer 50% | 412 | 43.5 (38.1-49.0) |
|  | Richer 50% | 551 | 56.5 (51.0-61.9) |
| Parity 1-2 (n=1325) | Poorer 50% | 647 | 46.5 (42.1-51.1) |
|  | Richer 50% | 695 | 53.5 (48.9-57.9) |
| Parity 3-4 (n=1036) | Poorer 50% | 533 | 49.5 (45.4-54.7) |
|  | Richer 50% | 524 | 50.5 (46.3-54.6) |
| Parity ≥5 (n=1132) | Poorer 50% | 636 | 57.8 (53.5-62.0) |
|  | Richer 50% | 458 | 42.2 (38.0-46.5) |
