# Supplementary material for: Inequity in uptake of hospital-based childbirth care in rural Tanzania: analysis of the 2015–16 Tanzania Demographic and Health Survey
Source: Health Policy Plan. 2021 Jul 19;36(9):1428–40. doi: 10.1093/heapol/czab079 (PMC8505858; doi:10.1093/heapol/czab079)
Supplement: czab079_Supp [file czab079_supp.zip › Supplementary_Table_2a_&_2b.docx]

*Supplementary Table 2a - Joint effect of SES and parity on adjusted OR of home versus primary care birth among rural women with a live birth in the preceding five years. DHS 2015-16 Tanzania (n=4456)*

|  | Adjusted OR (95% CI) | Adjusted OR (95% CI) | Adjusted OR (95% CI) | Adjusted OR (95% CI) |
| --- | --- | --- | --- | --- |
| Wealth group | Parity 0 | Parity 1-2 | Parity 3-4 | Parity ≥5 |
| 1 (poorest) | 0.66 (0.38-1.13) | 0.66 (0.38-1.13) | 0.97 (0.66-1.42) | ref |
| 2 (poorer) | 0.64 (0.36-1.12) | 0.92 (0.63-1.35) | **0.54 (0.36-0.82)** | 0.89 (0.61-1.28) |
| 3 (medium) | **0.49 (0.27-0.88)** | **0.57 (0.38-0.86)** | **0.43 (0.28-0.66)** | 0.78 (0.51-1.19) |
| 4 (wealthiest) | **0.43 (0.22-0.85)** | **0.41 (0.20-0.84)** | **0.23 (0.12-0.43)** | **0.29 (0.15-0.57)** |

*Supplementary Table 2b - Joint effect of SES and parity on adjusted OR of PHC birth versus hospital birth among rural women with a live birth in the preceding five years. DHS 2015-16 Tanzania (n=4456)*

|  | Adjusted OR (95% CI) | Adjusted OR (95% CI) | Adjusted OR (95% CI) | Adjusted OR (95% CI) |
| --- | --- | --- | --- | --- |
| Wealth group | Parity 0 | Parity 1-2 | Parity 3-4 | Parity ≥5 |
| 1 (poorest) | **8.03 (4.45-14.46)** | 0.62 (0.32-1.20) | 0.83 (0.47-1.49) | ref |
| 2 (poorer) | **0.49 (0.29-0.85)** | **3.03 (1.69-5.43)** | 0.59 (0.32-1.09) | 0.95 (0.57-1.59) |
| 3 (medium) | 0.67 (0.40-1.11) | **2.61 (1.49-4.56)** | 1.04 (0.57-1.89) | 0.89 (0.53-1.49) |
| 4 (wealthiest) | 0.91 (0.54-1.56) | **6.12 (3.37-11.11)** | 1.62 (0.84-3.12) | 1.35 (0.73-2.50) |
