# Supplementary figures and images for: Inequity in uptake of hospital-based childbirth care in rural Tanzania: analysis of the 2015–16 Tanzania Demographic and Health Survey
Source: Health Policy Plan. 2021 Jul 19;36(9):1428–40. doi: 10.1093/heapol/czab079 (PMC8505858; doi:10.1093/heapol/czab079)

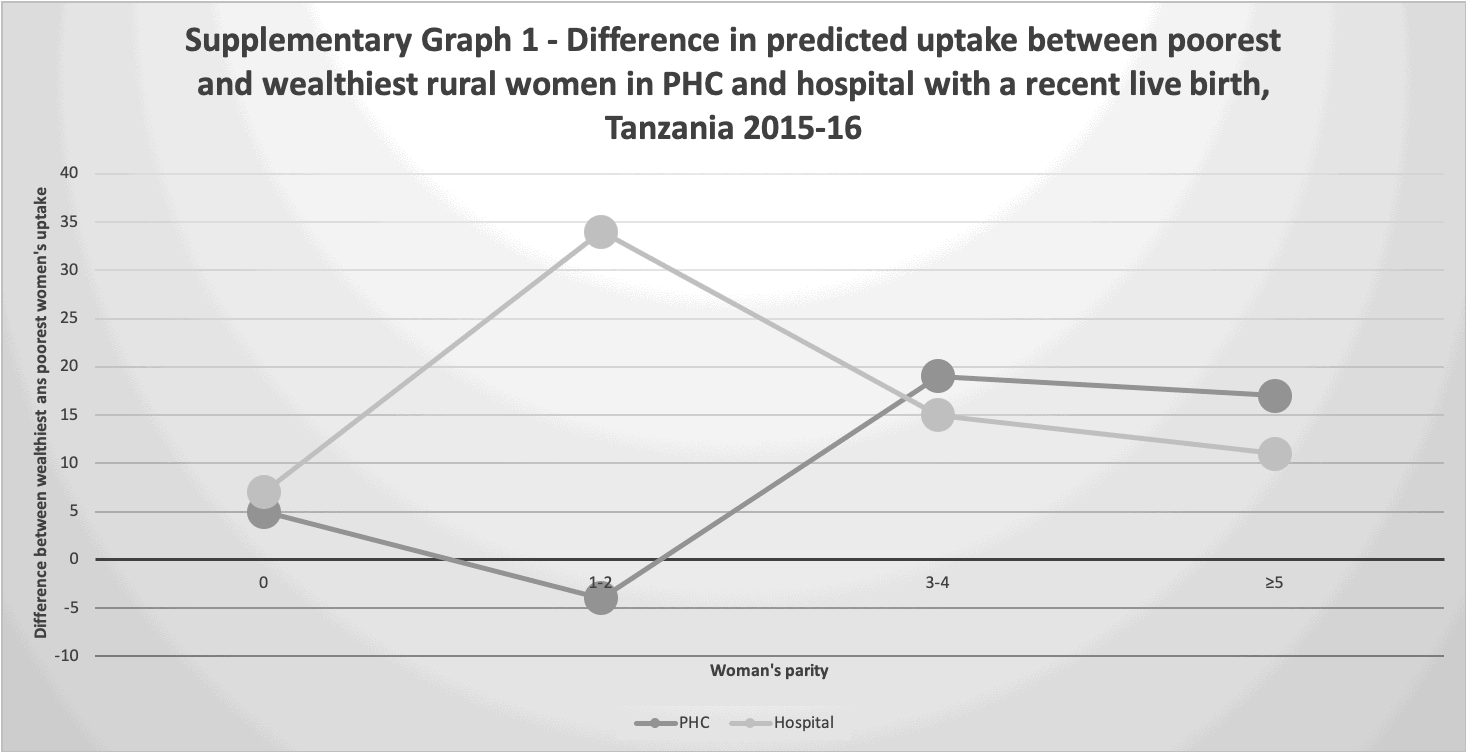

Supplement: czab079_Supp [file czab079_supp.zip › Supplementary_Graph_1.gif]
